# Theory and associated phenomenology for intrinsic mortality arising from natural selection

**DOI:** 10.1371/journal.pone.0173677

**Published:** 2017-03-29

**Authors:** Justin Werfel, Donald E. Ingber, Yaneer Bar-Yam

**Affiliations:** 1 Wyss Institute for Biologically Inspired Engineering, Harvard University, Cambridge, Massachusetts, United States of America; 2 New England Complex Systems Institute, Cambridge, Massachusetts, United States of America; 3 Harvard Medical School and Children’s Hospital, Boston, Massachusetts, United States of America; 4 School of Engineering and Applied Sciences, Harvard University, Cambridge, Massachusetts, United States of America; National Taiwan University, TAIWAN

## Abstract

Standard evolutionary theories of aging and mortality, implicitly based on assumptions of spatial averaging, hold that natural selection cannot favor shorter lifespan without direct compensating benefit to individual reproductive success. However, a number of empirical observations appear as exceptions to or are difficult to reconcile with this view, suggesting explicit lifespan control or programmed death mechanisms inconsistent with the classic understanding. Moreover, evolutionary models that take into account the spatial distributions of populations have been shown to exhibit a variety of self-limiting behaviors, maintained through environmental feedback. Here we extend recent work on spatial modeling of lifespan evolution, showing that both theory and phenomenology are consistent with programmed death. Spatial models show that self-limited lifespan robustly results in long-term benefit to a lineage; longer-lived variants may have a reproductive advantage for many generations, but shorter lifespan ultimately confers long-term reproductive advantage through environmental feedback acting on much longer time scales. Numerous model variations produce the same qualitative result, demonstrating insensitivity to detailed assumptions; the key conditions under which self-limited lifespan is favored are spatial extent and locally exhaustible resources. Factors including lower resource availability, higher consumption, and lower dispersal range are associated with evolution of shorter lifespan. A variety of empirical observations can parsimoniously be explained in terms of long-term selective advantage for intrinsic mortality. Classically anomalous empirical data on natural lifespans and intrinsic mortality, including observations of longer lifespan associated with increased predation, and evidence of programmed death in both unicellular and multicellular organisms, are consistent with specific model predictions. The generic nature of the spatial model conditions under which intrinsic mortality is favored suggests a firm theoretical basis for the idea that evolution can quite generally select for shorter lifespan directly.

## Introduction

Lifespan extension is a topic of broad interest as old as recorded history, with a succession of varied treatments advocated for those interested in living longer [1–4]. Lifespans vary widely among species [5–8] and can be experimentally altered by genetic modification [9–17] and selective breeding [18, 19]. This variability and plasticity suggests that lifespan should be a heritable trait and subject to selective forces. A first intuition might conclude that the characteristic lifespan for organisms of a given species evolves in a straightforward way to some optimum length that suits their particular ecological conditions [5]. However, further reflection points to a difficulty with this idea: the benefit of evolving a longer lifespan is clear, but under what conditions would selection favor an earlier death? It seems straightforward that a gene that contributes to or hastens the death of its bearer should always be selected against.

Standard evolutionary theory has long explained senescence (impaired function with increasing age) as the result of two core effects, both based on the observation that the strength of natural selection should decrease as age increases. Because sources of extrinsic mortality (predation, disease, accident, etc.) have more chances to kill an organism the longer it lives to be exposed to them, a population will typically have fewer older individuals than younger ones, even if there is no senescence. Thus, a genetic effect that is only expressed at older ages will affect a smaller part of the population, and the intensity of selection for or against it is weakened. The mutation accumulation theory [20, 21] describes traits with late-acting negative effects, which are only weakly selected against and hence not eliminated. The antagonistic pleiotropy theory [22] posits genes conveying both early-life benefits and later-life penalties; aided by the decreasing strength of selection with age, such genes can be directly favored by selection, providing a net benefit to their bearers. The disposable soma theory [23], often characterized as a special case of antagonistic pleiotropy, considers that organisms have a limited amount of resources to divide between maintenance and reproduction; anything devoted to repairing damage and prolonging one’s own life comes at the expense of producing offspring, and so a strain with shorter lifespan but greater fecundity can outcompete a longer-lived strain. These factors are generally considered to provide the complete basis for senescence and intrinsic mortality, while older ideas of evolution of direct lifespan control [5] are considered untenable. There is a broadly accepted understanding that evolution does not and *cannot* act directly to shorten or otherwise limit lifespan [2, 24, 25].

However, a variety of observed phenomena have been and/or remain difficult to reconcile with accepted frameworks [26]. One example is semelparity: organisms of some species reproduce only once and die after reproducing, and may live for a variable period [6] before the act of reproduction apparently triggers their death. Because programmed death is inconsistent with classic theories, other interpretations for semelparity have been sought; the traditional explanation is that so much energy goes into reproduction that none is left over for essential physiological maintenance afterwards [26]. However, cases such as octopus, where surgical removal of a gland after reproduction allows continued survival and resumption of copulation [27], are difficult to reconcile with this interpretation; such a case is particularly suggestive of an explicit programmed death mechanism, an interpretation rejected because the accepted theoretical understanding states that programmed death of an organism should in general not be possible.

Another example has to do with multicellularity versus unicellularity. It has been argued that in multicellular organisms—where the distinctions between gametes and other cells (germline versus soma), and between parent and offspring, are clear—senescence is inevitable, while unicellular organisms (lacking those distinctions) should be effectively immortal [5, 22, 24, 26, 28]. However, certain multicellular organisms have been reported to have negligible or even negative senescence, i.e., displaying insignificant or declining effects of aging according to population-statistical or physiological measures [6, 7, 29–37]. Conversely, senescence has been reported in organisms with no clear germ/soma distinction [38], and programmed cell death reported in unicellular organisms [39–42]. Such observations have been explicitly recognized as surprising and difficult to reconcile with traditional theories [26, 31, 38–40, 43, 44].

A third example centers on extrinsic mortality. It is widely reported as a key prediction of traditional theories that higher extrinsic mortality rates should result in the evolution of shorter intrinsic lifespans [22, 24, 26, 45–48]. Alternative theoretical analyses predict that if the extrinsic mortality rate is independent of age, it should have no effect on evolved lifespan [49]. Notably, a key empirical study observed that guppies that had evolved subject to higher levels of predation exhibited longer lifespans and lower rates of aging [48].

Other examples include large single-gene effects on lifespan [9] and other central regulators of aging [10, 15, 16]. Similarly, clinical interventions that delay or even reverse changes associated with aging have been demonstrated in laboratory animals [50, 51], suggesting the presence of active physiological control and a means to intervene in it.

The comparison of theoretical and empirical results, which appear contradictory, motivates important questions. Is it, after all, possible for selection to favor shorter lifespans, due to some benefit that shorter lifespan itself provides? What theoretical framework can yield results consistent with the general possibility of selecting lifespan control, and does it agree with specific empirical results that are available? What are the relevant and irrelevant features of a system that determine whether lifespan control is favored or opposed? Addressing these questions includes identifying how traditional theories give valid results for the conditions they consider, but may not apply without modification in other situations. The key is a recognition that under certain conditions, population averages are insufficient as a way of capturing a system’s full behavior. As a result, additional variables that are not contained in traditional analytic treatments become necessary for valid mathematical descriptions of these systems [52–54]. The issue particularly often arises for spatial systems, where heterogeneity (symmetry breaking) means that analyses based on averaging may predict qualitatively different behavior than the spatial system exhibits. The results of a theory that uses averaging apply to systems where homogeneity is a valid approximation, but may not apply otherwise. The importance of the heterogeneity that can develop in spatially distributed systems has become increasingly recognized in many areas of ecology and evolutionary biology [55–58].

In particular, recent work in spatial modeling of evolutionary systems has given new insight into altruistic behaviors of individuals within populations [59–61], with relevance to phenomena including reproductive restraint [62, 63], social communication [64], and sexual reproduction [65, 66]. Spatial systems, where persistent local effects are possible, have qualitatively different behavior from non-spatial ones, in which averaging over the system means that everything is effectively happening in the same place. Importantly, the traditional theories of senescence are implicitly based on non-spatial assumptions of averaging. In spatial evolutionary systems, unlike non-spatial ones, there can typically be a short-term advantage but long-term disadvantage (which may appear only after a time scale of many generations) incurred by overly exploitative variants [62, 64]. “Selfish” variants that outcompete their neighbors, at the expense of depleting their local environment, have an advantage on short spatial and temporal scales. However, on longer scales, their descendants are left in impoverished environments and can be outcompeted by others in richer areas. A longer-range view of “time-dependent fitness” [62] recognizes these effects, and predicts the broad success of altruism and individual restraint in spatial models.

Spatial evolutionary models have demonstrated in the past that an evolutionary response to selection favoring self-limited lifespan is not a theoretical impossibility [67–69], typically relying on limiting assumptions such as continual introduction of highly advantageous mutations [70], pre-existing senescence in the form of decreasing fecundity [71, 72] or decreasing competitive fitness [73] with increasing age, frequent deadly epidemics among physically and genetically close relatives [74], or explicit group selection among nearly-isolated subpopulations [75]. These assumptions can restrict how broadly these results can be considered to apply [76].

In a recent publication [77], using spatial evolutionary simulations, we demonstrated a generally applicable mechanism for the evolution of active lifespan control without such limiting assumptions. In this paper we review and extend the model introduced in [77] and its analysis, and discuss the application of the results to empirical observations. More specifically, in this paper, in order to better compare the analysis here with traditional population biology concepts as well as ecological observations, we present simulations evaluating reproductive success as a function of generation rather than time, enabling the comparison of strains with different lifespans; we present results exploring a much larger region of the parameter space of the model; we describe simulations incorporating extrinsic mortality, with relevance to empirical observations difficult to explain with established theories; we provide quantitative analysis of population structure; we include a more extensive discussion of model variants to better explain the wide applicability of the results, their biological relevance, and their quantitative results; we provide a more complete discussion of relevance of our results to empirical observations of biological systems, especially those which have posed difficulties for established theories; we discuss the time-dependent fitness framework that provides helpful insights into the reason for the violation of traditional theoretical results; and we provide a more complete discussion of the traditional perspectives on lifespan control as presented in the literature to clarify how this work builds upon prior work and does not contradict its findings when the assumptions used therein are satisfied.

The numerical simulations we discuss here and in [77] were designed to explore the mechanisms and conditions under which intrinsic mortality, a particularly extreme form of individual restraint, might be favored in spatial models. The results showed that intrinsic mortality is consistently favored, for a wide range of model assumptions and parameters, with a very strong advantage. The two conditions necessary for self-limited lifespan to be favored were limited dispersal and limited resources, both generic features of natural systems. The results are moreover consistent with a variety of empirical observations, including those noted above as having posed challenges for traditional theories of aging and mortality.

## Methods

We considered a family of explicitly spatial models of an interacting pair of organism types, in which one population lives at the expense of the second, as in predator-prey, pathogen-host, or herbivore-plant systems. Analytic treatments [77] average over or otherwise approximate the complex dynamic patterns that are responsible for the distinct behavior of spatial models. Pair approximations and higher-order moment closure techniques incorporate some spatial heterogeneity, and can identify effects not captured by a basic mean-field treatment [78–80]; however, they are not always sufficient to capture phenomena observed in fully spatial systems [79–81]. Thus, we relied primarily on direct simulation to characterize the behavior of these models.

The model (first introduced in [77]) used a stochastic cellular automaton to consider a population of abstract organisms (“consumers”) with limited but self-renewing resources (e.g., prey or hosts). Sites in a two-dimensional lattice represented empty space, available resources, or a consumer together with resources (a consumer could not occupy a site in the absence of resources). At each time step (synchronous update), resources alone reproduced into neighboring empty sites, with probability *g* for each empty site (resource growth); consumers reproduced into neighboring resource-only sites, with probability *p* for each resource site (consumer reproduction); consumers exhausted resources in their site, leaving empty space, with probability *v* per time step (consumer consumption) and *c* per reproduction (consumer reproduction cost); and consumers died due to intrinsic mortality, leaving resources, with probability *q* (consumer intrinsic mortality). A consumer’s mean intrinsic lifespan is *L* = 1/*q*. To explore the evolution of lifespan in the consumer population, the intrinsic death rate *q* as well as the reproduction rate *p* were heritable and independently subject to mutation: with probability *μ*_*q*_ (*μ*_*p*_), a consumer offspring has a value of *q* (*p*) differing from that of the parent by ±*ϵ*_*q*_ (±*ϵ*_*p*_) (positive or negative mutations with equal probability).

An alternative description of the model gives the probabilities of site transitions at each time step. The probability of an empty site becoming a resource-only site is $1-{\left(1-g\right)}^{N_{R}}$, where *N*_*R*_ is the number of resource-only sites among the site’s four neighbors. The probability of a resource-only site becoming a consumer site is $1-\prod_{i}^{N_{C}}\left(1-p_{i}\right)$, where *N*_*C*_ is the number of consumer sites among the site’s four neighbors, and *p*_*i*_ are the reproduction probabilities for the corresponding sites. This expression corresponds to each neighboring consumer site *i* having an independent probability *p*_*i*_ of trying to reproduce into the resource-only site in question; if more than one does so, one is chosen at random (with equal probability) to be the parent. The parent’s identity is relevant when assigning values of *p* and *q* to the new offspring, as well as affecting that parent’s probability of death during the time step: the probability of a consumer site becoming an empty site is *v* + *rc*, where *r* is the total number of offspring produced by that consumer during that time step. The probability of a consumer site *i* becoming a resource-only site is *q*_*i*_.

Two types of simulations investigated the evolution of lifespan control in a population. (1) In “ascendance” studies, we tracked the evolution of the intrinsic death rate *q* and reproduction rate *p* over time in a randomly initialized consumer population, to investigate what values dominate in the long term. In addition to the populations with intrinsic mortality with which this research is primarily concerned, we did studies with control populations without intrinsic mortality, matching models studied in earlier work [62–64], in which *q* was fixed at 0 for all consumers and only *p* evolves; these two types of populations were designated “mortal” and “immortal”, respectively. The ascendance studies discussed here extended those of [77] over a much larger region of the parameter space. (2) In “invasion” studies, we considered the ability of a single consumer to take over a population, in order to ask: If a rarely occurring mutation could confer or remove the capacity for lifespan control, would that mutant have an advantage or a disadvantage in its later spread through the population? These studies introduced one consumer into a steady-state population, followed its lineage until fixation (extinction of either invaders or invaded), and examined the probability of successful invasion in many such trials. For each set of parameter values, we performed these invasion studies for each of the four possible combinations where the invading and invaded populations were each mortal or immortal; for mortals invading populations of immortals, *q* was 0 for the initial invader, but potentially nonzero for descendants through mutation.

Simulations were performed on lattices of size 250 × 250 unless the consumer population was not stable (quickly going to extinction in such cases—primarily a problem for immortal populations). Accordingly, some simulations were run on larger arrays, each on the smallest of {250 × 250, 500 × 500, 750 × 750} for which a steady-state population of consumers could persist. Increasing the lattice size further does not change the steady-state values of *p* and *q*. For immortal consumers, we used:

- 250 × 250: *g* = {0.05, 0.1, 0.2}, *v* = {0.1, 0.2}
- 500 × 500: *g* = {0.05, 0.1}, *v* = {0.005, 0.01, 0.05}; *g* = 0.2, *v* = {0.01, 0.05}
- 750 × 750: *g* = 0.01, *v* = {0.005, 0.01}; *g* = 0.2, *v* = 0.005

In other cases (*g* = 0.005, *v* = {0.005, 0.01, 0.05, 0.1, 0.2};*g* = 0.01, *v* = {0.05, 0.1, 0.2}), immortal populations were found not to be stable even on arrays of 2000 × 2000 sites. Mortal populations were stable on 250 × 250 arrays in all cases, with the exception of *g* = 0.005, *v* = 0.2 which required a 750 × 750 array. Boundary conditions were periodic. Ascendance studies initialized the lattice randomly where each site was empty with probability 0.55, resource-only with probability 0.4, and consumer with probability 0.05, in the latter case with *p* chosen from a uniform distribution between 0 and 1 and *q* from a uniform distribution between 0 and 1 − *v* (starvation and intrinsic mortality were taken to be mutually exclusive possibilities). Invasion studies initialized the lattice with a configuration where the invaded population had been left for 2 × 10^5^ time steps to reach a steady state, and then chose one consumer at random to convert to an invader; 10^5^ trials were performed for each combination of mortal and immortal invading and invaded populations, or 5 × 10^5^ trials for the case of immortals invading mortals. If, during a run, a consumer offspring had a value of *p* (*q*) below 0 or above 1 (1 − *v*) due to mutation, that variable was set to the corresponding boundary value. To ensure that maintaining these boundaries did not introduce artifacts into the evolved values of *p* and *q* in ascendance studies (artificially pushing the mean values away from the boundaries), we adopted the following procedure for those studies: after an initial 10^5^ time steps to achieve steady state, the size of mutations *ϵ*_*p*_ and *ϵ*_*q*_ was gradually reduced over a period of an additional 10^5^ time steps (halving the values every 10^4^ steps), followed by a final 5 × 10^4^ steps over which the values of *p* and *q* were averaged to obtain the final steady-state values reported for ascendance studies. For simulations using this base model, we used *μ*_*q*_ = *μ*_*p*_ = 0.1275, *ϵ*_*q*_ = *ϵ*_*p*_ = 0.005, and *c* = 0. A number of variants to the base model explored other parameter values and model structure.

### Variants

As discussed in [77] and below, these numerical experiments had the unexpected and counterintuitive result that programmed mortality was strongly favored over immortality, without compensating individual benefit or other contributing factors like relative competitive advantage of more recently born consumers, for all parameter values tested. To ensure that this result was not an artifact of some assumption of the model, and to better understand the mechanisms and conditions that would produce the result, we created numerous variants of the base model and performed additional ascendance studies. These variants explored a variety of considerations relevant to the model’s applicability to natural and experimental systems, and clarified the robustness with which self-limited lifespan evolved. They included:

- increased cost of reproduction;
- explicit deterministic lifespan and
- other age-dependent senescence patterns, rather than a fixed probability of death per time step;
- local consumer mobility through rearrangement and/or migration;
- increased dispersal range of consumers or resources;
- resource depletion that is deterministic and gradual, rather than stochastic and binary;
- sources of extrinsic mortality;
- ability of consumers to adjust their rate of living in response to resource availability;
- spontaneous resource generation;
- consumers reproducing to replace neighbors in occupied sites;
- resources reproducing while exploited by consumers;
- sexual reproduction by consumers.

We describe each of these in turn.

#### Reproduction cost

When studying the evolution of restraint phenomena, it can be difficult to disentangle the contributions of multiple different factors to a result. For instance, consider simulations with immortal populations, which show the evolutionary success of reproductive restraint (*p* < 1) [62–64]; if the cost of reproduction is nonzero, one might interpret this restraint as having evolved in the service of conserving resources, to maximize direct individual reproduction. Choosing reproduction cost *c* = 0 helps clarify the mechanism: showing that restraint evolves even when reproduction cost is zero demonstrates that the above interpretation is incorrect. To separate the effects of reproduction cost, and following previous work [62–64], we focus in most simulation experiments on the limiting case of *c* = 0. However, since reproduction cost is of course nonzero in natural systems, in this variant we performed simulations testing nonzero values of *c*.

#### Deterministic lifespan

In this variant, the genotype specifies intrinsic lifespan directly as a fixed length of time: *L* time steps after a consumer is born, it dies, regardless of remaining resources. In one form of this variant, lifespan is fully deterministic in this way. Another form adds variability by choosing the lifespan from a normal distribution with mean at *L*.

#### Increasing mortality with age

In this variant, a consumer’s probability of death at each time step is given by the Gompertz equation [48] *m*(*t*) = *m*_0_*e*^*qt*^, where *m*_0_ is a constant, *t* is the number of time steps since the consumer’s birth, and *q* is a heritable value as in the base model.

#### Consumer migration

In this set of variants, we allow consumer mobility. For this purpose, we split each time step into three successive stages: first, reproduction of both resources and consumers; second, resource depletion and consumer death through both starvation and intrinsic mortality; and third, mobility, in which each consumer (asynchronously, in random order) is able to move. We tested various types of mobility: (a) a consumer trades places with a randomly chosen neighboring consumer (if any exist); (b) a consumer moves to a neighboring, unoccupied resource site if such exists; (c) a consumer moves to a neighboring, unoccupied resource site when it exhausts the resources in its own site, in order to avoid the starvation during that time step that would otherwise occur; (d) a consumer exchanges places with a nearby randomly chosen non-empty site of either type. We tested these variants with neighborhoods ranging from the four nearest neighbors to a 7 × 7 square centered at the consumer’s original position.

#### Increased consumer or resource dispersal

In this variant, consumers or resources are not limited to reproducing into only the four neighboring sites. For ease of implementation, the model was modified as follows. Instead of the base model’s synchronous update where each site was simultaneously updated based on its value and those of its neighbors, an asynchronous update was used in which sites were successively chosen in random order for updates of the following form: empty sites remained empty; resource sites remained resources, and had a probability *g* of also reproducing by converting one empty site to a resource site; consumer sites had a probability *v* of becoming an empty site and *q* of becoming a resource site (*v* + *q* ≤ 1), and separately a probability *p* of reproducing by converting one resource site to a consumer site. For such resource or consumer reproduction, the offspring site was chosen randomly from all sites of the appropriate type such that both the row and column indices differed by at most *R*_*R*_ or *R*_*C*_ (for resources and consumers, respectively) from those of the parent.

#### Continuous-valued resources and deterministic consumption

In this variant, we treat resources as continuous-valued and consumption as occurring with a fixed rate. All sites are characterized by the quantity of resources they contain, with a value from 0 to 1. Consumers deplete resources by an amount *v* per time step, and *c* per reproduction. When resources in a site reach 0, the consumer there dies. If the consumer dies prematurely due to intrinsic mortality, residual resources remain for future exploitation. When an empty site is converted to one containing resources, the resource value there is set to 1. In one form of this variant, partially depleted resources are left unreplenished; in other forms, partially depleted resources are replenished slowly or quickly over time (to a maximum of 1).

#### Extrinsic mortality

In this variant, we added a factor representing sources of extrinsic mortality (corresponding to deaths through, e.g., predation or accidents). Whereas in the base model the probability per time step that a consumer would die leaving resources is *q*, in this variant that probability is *q* + *Q*, with *Q* being the extrinsic mortality.

We did simulations with two forms of this variant. The first form used a single value of *Q* constant over the whole lattice, and investigated what values of *q* and *p* evolved in the consumer population in this environment, comparing them to the values that evolved in the absence of extrinsic mortality (*Q* = 0). The second form considered spatial variation in extrinsic mortality. In the natural environment in which the guppies of the study noted above [48] evolved, features of the landscape led to different levels of extrinsic mortality in different places: predators were excluded from reaches of streams above waterfalls. In an analogue to such settings, one in which movement of the species of interest is not restricted by such boundaries, we applied a different value of *Q* to each of four quadrants of a 500 × 500-site lattice. A consumer’s probability of death leaving resources is its own value of *q* plus the location-dependent value of *Q*; consumer spread through reproduction is not affected. The evolved value of *q* could then be measured for subpopulations sampled from within each quadrant.

#### Consumers can adjust rate of living in response to resource shortages

To consider the ability exhibited by many organisms to adjust rate of living in response to environmental conditions (e.g., dauer formation in *C. elegans*, dietary restriction [26, 82]), we explored a variant based on the continuous-valued resource variant described above. In this variant, if a consumer is in a site with resource value below a threshold *T*, it adjusts its consumption *v*, reproduction *p*, and intrinsic mortality *q* all by a multiplicative constant *k*. Additionally, depleted resources are gradually renewed, by an amount *D* per time step, in both resource-only sites and those occupied by consumers.

#### Spontaneously generated resources

In this variant, we consider spontaneous appearance of resources at any location in space. This model corresponds, e.g., to certain plant-herbivore systems, where a plant such as grass can be cropped down to its roots and regrow. Empty sites in this variant become resource sites with probability *g* at each time step independent of whether other resources are located nearby. Resources are accordingly generated much more readily than in the base model, and so a much lower value of *g* produces an overall level of resource production more comparable to that in the base model.

#### Reproduction supplants existing consumers

In the base model, existing consumers prevent reproduction by neighbors into the sites they themselves occupy, in effect perfectly defending their territories. The opposite extreme is for new challengers always to defeat older competitors. In this variant, we allow consumers to reproduce not only into sites with resources alone, but also those with consumers already present, replacing the previous consumer.

#### Resources reproduce even when exploited by consumers

In this variant, we explored the consequences of allowing resources to reproduce even when consumers are present—i.e., resource sites reproduce with probability *g* in the absence of consumers and *g*′ in their presence (in the base model, *g*′ = 0).

#### Sexual reproduction by consumers

In this variant, when a consumer reproduces, a second consumer is randomly chosen to be the other parent of the offspring, with the values of *p* and *q* for the offspring being the average of those of the two parents plus mutation as in the base model. In one form of this variant, the second parent is chosen from nearby; in another form, the second parent is chosen from the full consumer population.

### Long-term reproductive success

To evaluate the long-term reproductive success of different types, a modified verson of the invasion studies without mutation was used. The simulation was initialized with a steady-state configuration for given values of parameters *g*, *v*, *c*; all consumers had the corresponding equilibrium values of *p* and *q*, and mutation was turned off (*μ*_*p*_ = *μ*_*q*_ = 0). One consumer was chosen at random and converted to an invader, and its offspring followed as in the regular invasion studies above, with *q* set to a new value for invaders and *μ*_*q*_ still 0. Because strains with different lifespans may have generations of different lengths, studies were performed tracking the number of descendants of the initial invader in two different ways: as a function of time, and as a function of generation number. For the former, the initial invader was chosen from the entire consumer population; for the latter, the initial invader was chosen from the set of newly-born consumers, so that all its offspring were identified and assigned the correct generation number. Each consumer in the invading population has a generation counter, starting with 0 for the original single invader, and with each offspring’s counter being one higher than that of its parent. This allows reporting the number of descendants of an invader either at a given time (counting members of all generations alive at that time) or of a given generation (no matter when in time they live).

The number of invaders was recorded as a function of time and of generation number after invasion, until extinction of either the invaders or the original invaded type. This process was repeated 40000 times, and the number of invaders at each time and each generation number averaged across the 40000 invasions. Each such set of 40000 invasions constituted one run, giving a measure of the mean number of invaders after an invasion. The variance among multiple such runs provided a measure of the standard error.

### Non-spatial model

For more direct comparison between the base model and a non-spatial version, we can also consider the predictions of the latter explicitly, both through simulation and formal analysis.

In the non-spatial simulation, each site is updated based not on its few neighbors (as, by hypothesis, neighborhood relations no longer exist) but rather on the average occupancy of all sites throughout the system. In a simple form of this, we can take the probability of an empty site transitioning to a resource-only site to be *n*_*R*_*g*, where *n*_*R*_ is the fraction of sites in the resource-only state; the probability of a resource-only site transitioning to a consumer site is $\sum_{i}p_{i}/N=n_{C}\bar{p}$, where *N* is the total number of sites in the system, *n*_*C*_ is the fraction of sites occupied by consumers, and $\bar{p}$ is the mean reproduction rate in the consumer population; the probability of a consumer site transitioning to an empty site is *v*; and the probability of a consumer site *i* transitioning to a resource-only site is *q*_*i*_. (For simplicity, we take reproduction cost *c* = 0, as in most of the simulations using the base model.) Heritability and mutation occur as before: when a resource-only site transitions to a consumer site, one consumer from the population is chosen at random to be the parent, with probability weighted by *p*_*i*_; and the offspring has the values of *p*_*i*_ and *q*_*i*_ of the parent, with probability *μ*_*p*_ (*μ*_*q*_) differing by *ϵ*_*p*_ (*ϵ*_*q*_). As in the base model, *p* and *q* are prevented from mutating beyond the limits of their meaningful ranges, and the mutation sizes *ϵ*_*p*_ and *ϵ*_*q*_ are reduced over time to prevent this thresholding from introducing artifacts in the evolved values. Initialization is likewise identical to the base model. This non-spatial model can be used in ascendance studies to examine its predictions about the evolution of *p* and *q*.

Eliminating mutation allows an analytic formulation that makes predictions about invasions [77]. Suppose first that there is only one consumer strain, with parameter values {*p*, *q*}. At steady-state, the rate at which sites make transitions to the consumer state equals the rate at which they make transitions away from it: *n*_*R*_*n*_*C*_*p* = *n*_*C*_(*v* + *q*). This gives the steady-state fraction of resource-only sites, as a function of *p* and *q*: *n*_*R*_ = (*v* + *q*)/*p*. Now add an invader with parameter values {*p*′, *q*′}. The site transition rate into this new consumer state is *n*_*R*_*n*_*C*′_*p*′, and the transition rate away is *n*_*C*′_(*v* + *q*′). Thus if (*v* + *q*′)/*p*′ < *n*_*R*_ = (*v* + *q*)/*p*, the frequency of the invader in the population will increase, while if (*v* + *q*′)/*p*′ > (*v* + *q*)/*p*, the invader will be eliminated. The implications of this calculation are discussed below.

## Results

**Fig 1:**
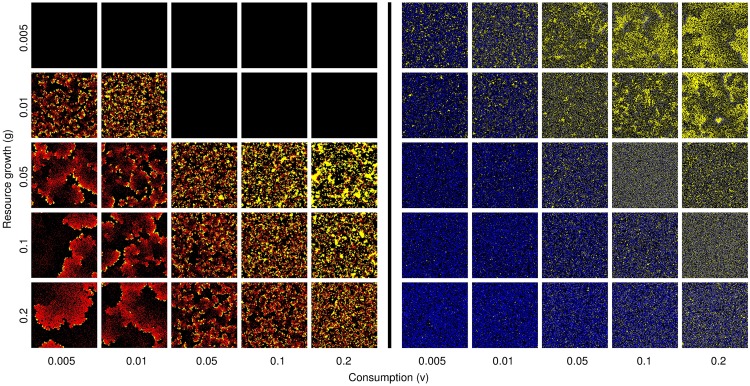
Model snapshots showing different spatial distributions of consumers and resources. Resources are shown in yellow; consumers with intrinsic mortality (nonzero *q*) are shown in blue, those without it (*q* fixed at 0) in red; empty spaces are shown in black. The values of *p* and *q* for each panel are those which evolve in ascendance studies (Fig 2B and 2C). Each panel shows 250 × 250 sites; parameter values for which populations without intrinsic mortality were not found to be stable are shown as empty lattices (details and full lattice sizes in Methods).

**Fig 2:**
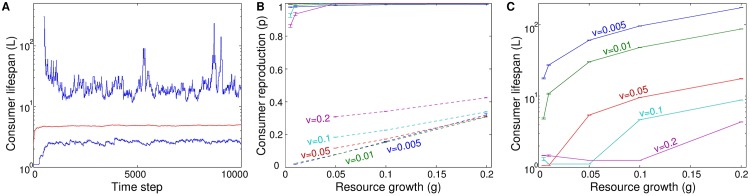
Ascendance studies show the consistent success of self-limited lifespan. (A) History of evolving consumer lifespan in one example simulation (*g* = *v* = 0.1, *c* = 0), showing population mean/maximum/minimum. (B,C) Steady-state average values of (B) consumer reproduction probability *p* and (C) intrinsic lifespan *L* = 1/*q*, for different values of parameters *g* and *v*. Solid lines show results for populations with intrinsic mortality (*q* free to evolve), dashed lines show results for populations without intrinsic mortality (*q* fixed at 0).

The ascendance studies with the base model showed that self-limited lifespan was consistently favored, for all parameter values tested. Changing the values of the constant parameters *g* and *v* changed the population size and distribution of consumers and resources (Fig 1) and specific equilibrium value of lifespan that evolved (Fig 2C); but the qualitative result, an evolutionary response to selection favoring lifespan limitation, was found in all cases.

The capacity for intrinsic mortality changed the characteristic population structure, as qualitatively visible in Fig 1. Table 1 shows quantitatively that the mean patch size (defined as the number of consumers in a connected set) was consistently larger for populations without intrinsic mortality than for those with it, for the same values of the ecological parameters {*g*, *v*}.

**Table 1:** Intrinsic mortality changes self-organized population structure.

		*v* = 0.005	*v* = 0.01	*v* = 0.05	*v* = 0.1	*v* = 0.2
*g* = 0.005	Immortal	N/A	N/A	N/A	N/A	N/A
	Mortal	8.7 ± 0.4	7.2 ± 0.4	1.01 ± 0.01	2 ± 1	2.0 ± 0.1
*g* = 0.01	Immortal	25 ± 1	12.5 ± 0.3	N/A	N/A	N/A
	Mortal	8.2 ± 0.3	7.9 ± 0.3	1.01 ± 0.01	1.02 ± 0.01	2.0 ± 0.5
*g* = 0.05	Immortal	250 ± 50	79 ± 8	12.3 ± 0.5	8.2 ± 0.7	6.3 ± 0.4
	Mortal	11.8 ± 0.4	11.2 ± 0.6	7.8 ± 0.4	1.08 ± 0.03	1.03 ± 0.01
*g* = 0.1	Immortal	700 ± 300	230 ± 50	24 ± 1	11.6 ± 0.9	7.4 ± 0.7
	Mortal	20 ± 1	18 ± 1	12.2 ± 0.5	8.1 ± 0.3	1.07 ± 0.03
*g* = 0.2	Immortal	5000 ± 4000	700 ± 300	50 ± 4	21 ± 2	10.5 ± 0.6
	Mortal	54 ± 5	46 ± 6	28 ± 2	17 ± 1	8.5 ± 0.2

Mean consumer patch size, averaged over all consumers, for different values of resource growth rate *g* and consumption rate *v*, and for populations with and without intrinsic mortality (“mortal” and “immortal”, respectively).

**Fig 3:**
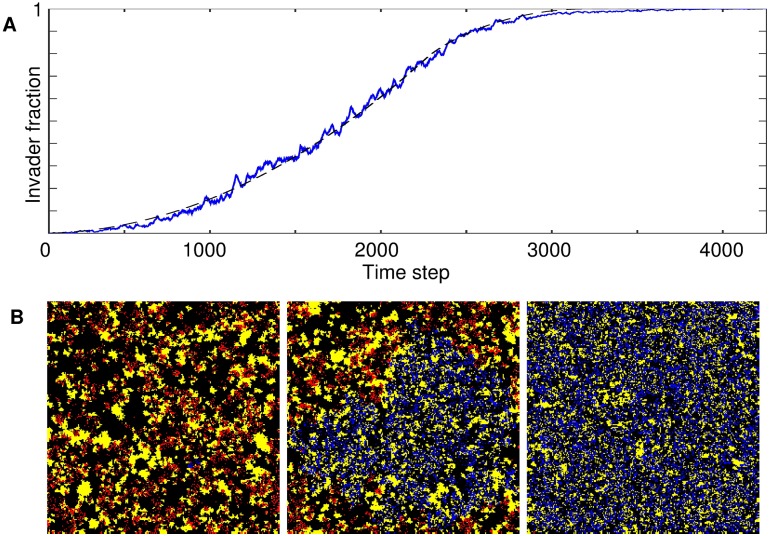
In a successful invasion of consumers without intrinsic mortality (*q* fixed at 0) by those with the capacity for intrinsic mortality (*q* = 0 for the initial invader, but potentially nonzero for descendants through mutation), the invader population spreads steadily. (A) The fraction of invaders in the population (solid line) increases almost monotonically with time. The process can be well approximated as if a boundary between invader- and invaded-dominated areas moves radially outward from the site of initial invasion with constant speed: the dashed line shows the fraction of the lattice area occupied by a circle whose radius increases linearly with time (periodic boundary conditions); the correlation between the invader fraction and the circle-area fraction (solid and dashed lines) is *r* = 0.999. Resource growth *g* = 0.05, consumer consumption *v* = 0.1. (B) Snapshots at 100, 2000, and 4000 time steps (colors as in Fig 1).

The invasion studies (Fig 3) showed a very strong advantage to the capacity for intrinsic mortality (Table 2, S1 Table) [77]. Consumers with this capacity (*q* = 0 for the invader, but potentially nonzero for its descendants through mutation) invading populations of consumers without intrinsic mortality (*q* fixed at 0 for all descendants) had a success rate typically two to three orders of magnitude greater than that of invaders without intrinsic mortality, across all values of the ecological parameters *g* and *v* tested. Conversely, consumers with no intrinsic mortality managed no successful invasions of populations with programmed death, for any values of *g* and *v*, in a total of 4.5 million trials.

**Table 2:** The capacity for intrinsic mortality strongly increases the probability of successful invasion.

	*v* = 0.05	*v* = 0.1	*v* = 0.2
*g* = 0.05	1.3 × 10^3^	1.6 × 10^2^	4.4 × 10^1^
*g* = 0.1	1.3 × 10^3^	1.7 × 10^2^	1.6 × 10^2^
*g* = 0.2	7.5 × 10^2^	2.2 × 10^2^	3.0 × 10^2^

Ratio of probabilities of successful invasion when populations of consumers with no capacity for intrinsic mortality (*q* = 0) are invaded by consumers with vs. without that capacity. Each entry in the table gives, for the corresponding values of *g* and *v*, the empirically observed probability of successful invasion if the invader has the capacity for intrinsic mortality (*q* = 0 for the initial invader, but potentially nonzero for descendants through mutation), divided by the probability of successful invasion if the invader lacks that capacity. S1 Table gives the full results for successful invasion probabilities in all cases, as well as those where the invaded population has intrinsic mortality.

The outcome with nearly all model variants was likewise the evolution of self-limited lifespan (Table 3). The exceptions (discussed next) were consistent with this understanding and with previous work: When consumer dispersal was large compared to the size of the space, the system was effectively well-mixed and lifespan control did not evolve. When resource availability was so great as to support the maximum amount of consumption and reproduction the model allowed, the system evolved to these limits. In natural systems, both dispersal and resources are normally limited.

**Table 3:** Evolved trait values for selected model variants and conditions tested.

Variant	*g*	*v*	*p*	*q*	Notes
Reproduction cost	0.05	0.05	1	0.150 ± 0.002	*c* = 0.05
	0.05	0.05	0.980 ± 0.007	0.052 ± 0.001	*c* = 0.4
Continuous-valued resources	0.05	0.05	1	0.2770 ± 0.0009	–
Deterministic lifespan	0.05	0.05	1	*L* = 12.0 ± 0.1	–
Senescence given by Gompertz equation	0.05	0.05	1	0.150 ± 0.003	*m*_0_ = 0.1 (Gradual senescence)
	0.05	0.05	1	−0.229 ± 0.001	*m*_0_ = 0.5 (Negative senescence)
Consumer rearrangement	0.05	0.05	1	0.053 ± 0.001	–
Consumer migration	0.05	0.05	0.984 ± 0.004	0.0316 ± 0.0004	–
Rearrangement + migration	0.05	0.05	0.96 ± 0.01	0.053 ± 0.001	7 × 7 neighborhood
Spontaneously generated resources	0.005	0.05	1	0.95	–
Reproduction supplants existing consumers	0.05	0.05	1	0.40 ± 0.05	–
Increased consumer dispersal	0.05	0.05	0.985 ± 0.005	0.24 ± 0.01	*R*_*R*_ = 1, *R*_*C*_ = 1
	0.05	0.05	0.934 ± 0.006	0.16 ± 0.01	*R*_*R*_ = 1, *R*_*C*_ = 2
Increased resource dispersal	0.05	0.05	1	0	*R*_*R*_ = 5, *R*_*C*_ = 1
	0.05	0.1	1	0.11 ± 0.01	*R*_*R*_ = 5, *R*_*C*_ = 1
	0.05	0.2	1	0.402 ± 0.006	*R*_*R*_ = 5, *R*_*C*_ = 1
Resources reproduce when exploited	0.05	0.23	1	0.25 ± 0.03	*g*′ = *g*
	0.23	0.23	1	0.231 ± 0.002	*g*′ = 0
	0.05	0.05	1	0.0103 ± 0.0002	*g*′ = *g*/3
	0.05	0.05	1	0	*g*′ = *g*/2
Sexual reproduction	0.1	0.2	0.715 ± 0.004	0.087 ± 0.001	Other parent chosen from 7 × 7 neighborhood
	0.1	0.2	1	0	Other parent chosen from entire population
Consumers adjust rate of living	0.1	0.2	1	0.342 ± 0.001	*D* = 0.05, *k* = *T* = 0.5
Base model	0.05	0.05	1	0.1847 ± 0.0005	–

When resources are limited and dispersion is local, *q* consistently evolves to a value significantly greater than 0. Uncertainty measurements refer to standard error of the mean, based on 10 independent experiments. See text for details of variants.

Increasing the consumer dispersal range *R*_*C*_ can disrupt the local neighborhood relationships that make it possible for evolution to lead to restraint. Large enough *R*_*C*_ makes the system effectively well-mixed. In such cases consumption increases through unopposed selection for faster reproduction and longer lifespan, until the consumer population exhausts all available resources and goes extinct. The dispersal range above which this occurs is a function of the ecological parameters *g*, *v*, *c* as well as the size of the simulation space. This is because *g*, *v*, *c*, *R*_*C*_ affect the length scale of the characteristic population structure; if that length scale is too large compared to the space, the spatial nature of the model breaks down. Thus a given value of *R*_*C*_ can result in extinction on lattices of a given size but allow evolution of restraint and a sustainable consumer population on larger lattices. For instance, with *g* = *v* = 0.05 and *c* = 0, a consumer population with *R*_*C*_ = 1 consistently goes extinct on a 50 × 50 lattice (in mean 5800 time steps), but survives (i.e., persists for at least 250000 time steps, evolving intrinsic mortality) on a 100 × 100 lattice; *R*_*C*_ = 2 consistently gives extinction on a 200 × 200 lattice (in mean 60000 time steps) but survival on a 250 × 250 lattice; *R*_*C*_ = 3 results in consistent extinction on a 250 × 250 lattice (in mean 5400) time steps), survival in 3 of 10 trials on a 500 × 500 lattice (with extinction in mean 100000 time steps in the other trials), and consistent survival on a 750 × 750 lattice. The values reported in Table 3 give results for 250 × 250 lattices (for which we did not observe extinction for the values of *R*_*C*_ reported there), averaging *p* and *q* over the last 50000 time steps of trials lasting 250000 time steps, as with all other entries in that table.

Increasing the resource dispersal range *R*_*R*_ increases the extent to which consumers generally have resources available, which affects the extent to which intrinsic mortality is favored. For instance, with *R*_*R*_ = 5, choosing *g* = *v* = 0.05 results in enough resource availability that intrinsic mortality is not favored; increasing the consumption rate *v* to 0.1 reduces resource availability and returns the model to the regime where intrinsic mortality is favored, and increasing *v* to 0.2 results in the evolution of still higher intrinsic mortality rates.

Spontaneous generation of resources greatly increases the level of resources available to consumers, so that for high values of *g*, the consumer population is not limited by resource availability and lifespan control does not evolve. Lower values of *g* result in lifespan control evolving.

When exploited resources reproduce (*g*′ nonzero), the overall level of resource availability increases. Hence this variant requires reducing *g* and/or increasing *v* to obtain results quantitatively comparable to those of the base model. For example, for *g* = *g*′ = 0.05, *v* = 0.23, the steady-state value of *q* is close to that of the base model for *g* = *v* = 0.05 and for *g* = *v* = 0.23. For a given *g* and *v*, increasing *g*′ leads to lower steady-state values of *q* (longer lifespan), just as increasing *g* for fixed *v* does in the base model (Fig 2). It is possible in this variant, if the level of resource growth is high enough compared to *v*, for consumers to have little enough impact on the spatial distribution of resources that evolution does not limit reproduction probability (for immortals) or lifespan (for mortals). For example, with *g* = *v* = 0.05, reproduction and lifespan limits occur for *g*′ ≤ *g*/3 and not for *g*′ ≥ *g*/2.

With sexual reproduction, the way the second parent is chosen determines whether self-limited lifespan evolves. When the second parent is chosen from nearby (e.g., from a 7 × 7 region centered on the offspring), finite lifespan is favored, as is reproductive restraint: *q* evolves to a value significantly greater than 0, and *p* to a value less than 1. When the second parent is drawn from the entire consumer population (i.e., a form of global dispersal), no restraint evolves: *q* evolves to 0 and *p* to 1.

**Fig 4:**
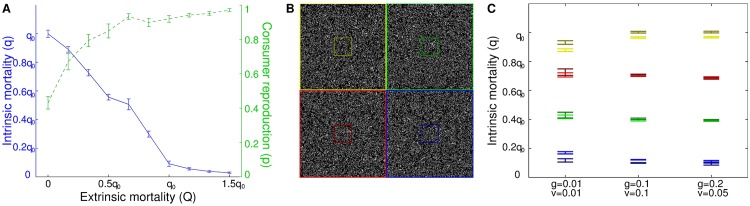
Increased extrinsic mortality results in lower intrinsic mortality. (A) Adding an additional source of extrinsic mortality of magnitude *Q* decreases the evolved value of intrinsic mortality *q*, and increases evolved reproduction probability *p*. The unit of measure *q*_0_ is the mean value of *q* that evolves for *Q* = 0;*g* = *v* = 0.01, *c* = 0.4. (B) In studies with spatially varying extrinsic mortality, different values of *Q* are imposed on consumers in different quadrants of the lattice: yellow, *Q* = 0; red, *Q* = 0.3*q*_0_; green, *Q* = 0.6*q*_0_; blue, *Q* = 0.9*q*_0_. Evolved *q* is evaluated based on sampling the consumer population two different ways: all consumers within each quadrant, and only those within a smaller region at the center of each quadrant (darker outlines) to avoid effects at the boundaries between quadrants. (C) Higher extrinsic mortality leads to lower evolved intrinsic mortality, for various values of *g* and *v* (*c* = 0). Sampling from whole quadrants vs. small central regions shows that boundary effects are minor in these simulations. Colors indicate sampling from the corresponding regions in (B). All error bars show the standard error of the mean from ten independent trials.

Adding extrinsic mortality reduced the evolved level of intrinsic mortality *q*, such that the total mortality level for a given set of parameters remained approximately constant (Fig 4A). Increasing extrinsic mortality could also increase the evolved value of reproduction probability *p* (Fig 4A). In studies with spatially varying extrinsic mortality, consumer subpopulations (as defined solely by how and where extrinsic mortality was imposed, not by any separation or restriction on reproduction distinct from the base model) similarly evolved lower intrinsic mortality in areas subject to higher levels of extrinsic mortality (Fig 4B and 4C).

The variant using the Gompertz equation can predict the evolution of different senescence patterns, depending on the parameter values. For instance, if intrinsic mortality is set to be initially higher than the equilibrium found in the base model, then negative senescence evolves.

**Fig 5:**
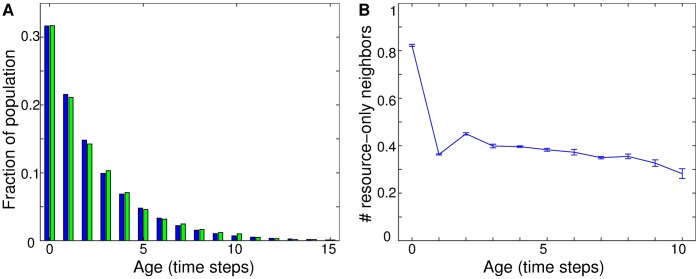
An age-structured view of the consumer population shows that differences in timing of reproduction occur between spatial and non-spatial models, despite identical population age structures and no explicit age-dependent differences in individual fecundity. (A) The distribution among age classes is unchanged between the spatial (blue) and non-spatial (green) models. (B) In the spatial model, consumers have a richer environment and hence more opportunities to reproduce in their first time step than at older ages. In the non-spatial model, consumers have the same number of neighboring resource sites at all ages, by definition. Resource growth *g* = 0.1, consumer consumption *v* = 0.1, reproduction cost *c* = 0; intrinsic mortality *q* = 0.215, reproduction probability *p* = 0.9991 (i.e., the equilibrium values in the spatial model of *q* and *p* for these values of *g*, *v*, *c*); no mutation (*μ*_*p*_ = *μ*_*q*_ = 0); error bars show the standard error of the mean from ten independent trials.

An age-structured view of the consumer population (Fig 5) shows that the spatial structure can give rise to changes in the timing of reproduction. In spatial and non-spatial populations with the same parameter values (*g*, *v*, *p*, *q*, *c* all held constant), the distribution of the population among age classes is the same in both versions of the model (Fig 5A). Moreover, in the base model, the reproduction probability *p* is constant at all ages (no reproductive senescence). These considerations show that the difference between spatial and non-spatial models, regarding whether self-limited lifespan evolves, is not due to differences in age structure. However, the shaping of the resource environment by the consumers has the result that in the spatial model, more opportunities to reproduce occur on average earlier in the consumer life history (Fig 5B). This observation is related to a result from traditional analytical treatments, which have showed that in stationary or growing populations, early fecundity is favored as an individual-level adaptation [21, 83]. The spatial model shows that, separately from any genetic control over the timing of reproduction, a shift to early reproduction can also occur as an outcome of the environmental feedback.

**Fig 6:**
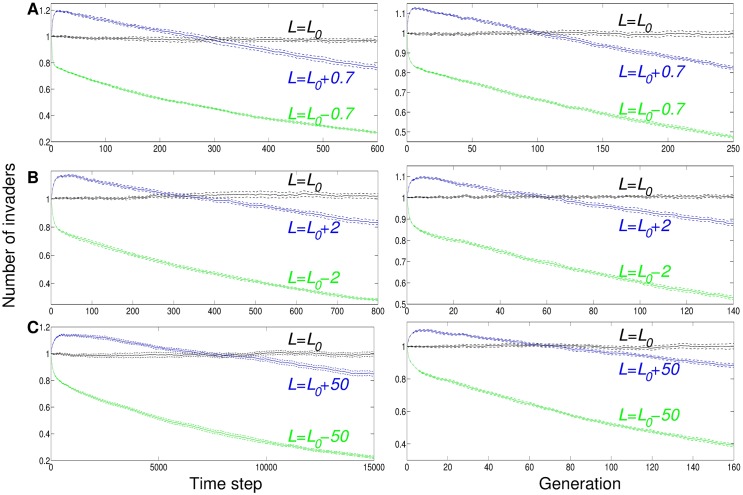
Longer lifespan can give short-term advantage and long-term disadvantage in spatial models. Plots show relative reproductive success (number of descendants) of individual mutants with lifespan *L*, introduced into a steady-state population with equilibrium lifespan *L* = *L*_0_ (Fig 2C), as a function of time (left) and of generation number (right). Parameter values are (A) *g* = 0.13, *v* = 0.2, *L*_0_ = 2.7, (B) *g* = 0.17, *v* = 0.1, *L*_0_ = 7.6, (C) *g* = 0.2, *v* = 0.005, *L*_0_ = 170, with resource growth rate *g*, consumption rate *v*, reproduction cost *c* = 0, and no mutation (*μ*_*p*_ = *μ*_*q*_ = 0) after the initial introduction. Error bars show the standard error of the mean among ten independent runs, each recording the mean for a set of 40,000 invasions.

The curves of reproductive success as a function of time and of generation (Fig 6) are qualitatively similar, and show that in comparison to a strain with equilibrium lifespan, a shorter-lived strain is outcompeted at all time scales, while a longer-lived strain has a reproductive advantage that may persist for dozens or hundreds of time steps or generations but is eventually outcompeted by the equilibrium strain.

The non-spatial model, in contrast to the spatial ones, predicted that higher *p* and lower *q* are always favored. The non-spatial ascendance studies showed that mean *p* in the consumer population evolves to the maximum possible value of 1 and mean *q* evolves to the minimum possible value of 0. The non-spatial invasion analysis shows that an invasion of a mutant with parameter values {*p*′, *q*′} in a population with values {*p*, *q*} will succeed if (*v* + *q*′)/*p*′ < (*v* + *q*)/*p*, and fail if (*v* + *q*′)/*p*′ > (*v* + *q*)/*p*—that is, for two strains with equal values of *q*, the one with higher *p* (greater probability of reproduction) will always be favored, and for two strains with equal values of *p*, the one with lower *q* (longer lifespan) will always be favored. These predictions match those of classic theories, which are based on individual reproductive success.

## Discussion

The model demonstrates that heterogeneity of limiting resources and self-organizing population structures lead to an adaptive self-limitation of lifespan (Fig 2), without direct compensating benefit to the individual. The tradeoffs that ultimately result in greater net reproduction are imposed through an extrinsic mechanism of long-term environmental feedback, which occurs as a result of the spatial structure. Strains with shorter lifespan shape their environments to be richer in resources, which their descendants can take advantage of [77]. Conversely, while a richer environment may facilitate reproductive success, shorter lifespan necessarily means less time in which to reproduce: increasingly shorter lifespans are not favored without limit. The balance between the two factors leads to the particular equilibrium value of intrinsic lifespan that demonstrates greatest success in the long run [77].

Importantly, the net reproductive benefits to self-limited lifespan via this mechanism may not appear for many generations (Fig 6); on shorter timescales, lifespan limitation conveys a net reproductive cost to its bearer. Thus, the mechanism is different from the standard evolutionary genetic theories founded on mutation accumulation and antagonistic pleiotropy. Such earlier models that predict an evolutionary response to selection favoring increased mortality account for it through intrinsic physiological tradeoffs: e.g., by putting energy into reproduction at the expense of maintenance, an individual may achieve earlier reproductive increases that more than compensate for lost later opportunities. In contrast to these classic mechanisms, which are based on selection directly on the reproductive success of individual organisms, the mechanism based in spatial heterogeneity predicts the evolution of traits that may become advantageous only for distant descendants. Thus, there is no compensating reproductive benefit to the individual of the shortened lifespan, as there would have to be in a non-spatial (well-mixed) model. Our results show that the benefit that makes shorter life favorable need not vest through reproductive success of the individual, but can do so rather through far descendants.

Moreover, the model variants show that an evolutionary response to selection favoring lifespan limitation occurs robustly, across many changes in model details and assumptions. The mechanism is not a rare phenomenon applying only under special conditions [76], but rather the generic result in spatial environments when resources are not unlimited.

The simulation results provide theoretical support for the idea that explicit lifespan control is consistent with natural selection, and that programmed mortality and senescence may act as mechanisms for achieving this control. Previous theoretical work has predominantly focused on and been successful at characterizing the spatially averaged case. It has concluded that lifespan control for its own sake cannot be selected for. Our study confirms this result for the spatially averaged case, and extends the analysis to show that different results apply in spatial systems with symmetry breaking. The appearance of contradiction with prior theory is thus due to the fact that we are considering a different case. This extension enables reconciling the theory of lifespan that has been developed up until now with the empirical results that suggest that lifespan control is possible. The mutation accumulation and antagonistic pleiotropy theories provide established mechanisms contributing to gradual senescence; the process explored here provides an additional mechanism, not a replacement—where multiple mechanisms are possible in biology, typically all may play a role. Our results suggest the broad applicability of an additional mechanism missing from previous theories. This mechanism can provide additional interpretations and novel understanding of a variety of observations in nature.

The apparently anomalous observations discussed in the Introduction can be interpreted naturally if explicit lifespan control and programmed death are admitted as explanatory mechanisms. Semelparity in general, and cases like the octopus in particular, are straightforward to interpret as involving a programmed death mechanism. Negligible and negative senescence in multicellular organisms, senescence in organisms with no clear germ/soma distinction, and programmed cell death in unicellular organisms are straightforward to explain if mortality and senescence are determined by selection, with favored lifespan set according to ecological conditions regardless of whether organisms are single-celled or multicellular. The spatial models predict that negligible or negative senescence may be favored under appropriate ecological conditions; conversely, programmed death can be favored for unicellular consumers as easily as for multicellular ones. The result of the study showing longer lifespans in guppies subject to higher predation is predicted by the spatial model (Fig 4); additional extrinsic mortality reduces the need for intrinsic mortality [84].

The potential uncoupling of intrinsic mortality and multicellularity has implications for our understanding of evolutionary history. Programmed cell death, critical during development in multicellular organisms (morphogenesis), has traditionally been viewed as having multicellularity as a prerequisite—the sharp distinction between somatic and germ cells intuitively enables some of the former to die without compromising their genetic contribution to the next generation—and accordingly it has been argued to have arisen contemporaneously or later [28, 85, 86]. Our results suggest that programmed cell death likely arose first in unicellular populations [43, 87–90] and later become co-opted for morphogenesis, perhaps helping to enable the evolutionary appearance of multicellularity. Similarly, the reproductive senescence observed in some unicellular organisms [6] could have been an exaptation for the cessation of division in somatic cell lines.

Other non-spatial analyses consider related although distinct issues. Age-structured population models [44, 91] examine the evolution of mutations affecting lifespan in large, homogenous populations. Studies of systems in which fitness is a function of population density characterize interactions between evolutionary and population dynamics [92]. Niche construction [93, 94] is a perspective on how organisms change the environment they inhabit in a feedback loop that affects their own survival and further evolution. Niche construction is traditionally treated in the spatial average: i.e., the organisms (considered as a homogenous group) effect a change to their environment (considered as a homogenous condition) and are in turn affected uniformly. Because these perspectives as they are normally formulated do not study the effects of spatial resource heterogeneity, i.e., the distinction between persistent impoverished vs. rich environments, they produce qualitatively different results from those presented here, as the spatial averaging eliminates the effect we describe; whether or not environmental feedback exists, a well-mixed environment always results in longer lifespan being favored. Adding a spatial aspect to studies following these other perspectives could similarly reveal unexpected novel outcomes.

It is sometimes raised as an objection to hypotheses of programmed aging that while many genes have been found which have large effects on lifespan, none has been found which eliminates aging altogether [26]. However, if self-limited lifespan has as powerful an adaptive advantage under as general conditions as our results suggest, it is to be expected that the genetic basis for it would be solidly and robustly established, with mechanisms for flexibly tuning lifespan to adjust to changing conditions but no simple way of eliminating the control altogether.

The idea that evolution can (and does) act directly to produce shorter lifespans goes back to at least 1870 with August Weismann, who argued that “duration of life is really dependent upon adaptation to external conditions …and that it is determined by precisely the same mechanical process of regulation as that by which the structure and functions of an organism are adapted to its environment” [5]. His reasoning explicitly relied on the idea that this adaptation occurred for the good of the species. Because of the untenability of the latter idea, the former was later strongly rejected (and continues to be rejected today); instead, theories based on individual self-interest were developed, in keeping with a position that explanations relying on some form of group selection should always be rejected if alternatives exist [22, 95] (see also further discussion below). Analytic [77] and experimental [96] studies that take a spatially averaged or well-mixed approach support the conclusions of the now-standard theories. For instance, experiments breeding fruit flies over many generations in spatially mixed populations showed that imposing higher extrinsic mortality resulted in the evolution of higher intrinsic mortality rates and shorter lifespans, in keeping with the predictions of classic theories [96]; non-spatial models correctly predict the outcomes of experiments in non-spatial systems. However, such spatially averaged systems exhibit qualitatively different behavior from systems which, like the natural world, possess spatial extent. In particular, spatial systems routinely demonstrate altruistic behaviors [59, 62–64, 97] which are not evolutionarily stable in spatially averaged models [77, 98, 99] or well-mixed laboratory populations [97]. This study is part of a growing body of work showing that selection above the level of the individual can have important effects on evolutionary outcomes, helping to understand evolved traits and altruistic behaviors opposed to direct individual self-interest [62–64, 100–103].

The robustness with which our simulations produce self-limited lifespan as the successful evolved outcome, under so many kinds of changes to the model structure, suggests that an evolutionary response to selection in favor of shorter lifespan and genetically programmed senescence may indeed be a quite general phenomenon. As such, it may have acted on the ancestors of human beings, with strong implications for human medicine. If aging is a functional adaptation, rather than a collection of inevitable breakdowns or genetic tradeoffs, then effective health and life extensions through dietary, pharmacological, or genetic interventions [50, 51, 104–111] are likely to be possible, with potential for significant impact (e.g., altering two genes extends nematode lifespan fivefold [10]). The effects of aging may one day be treated through manipulation of an underlying mechanism rather than as disparate symptoms. The fact that theoretical understanding of evolution can play a critical role in guiding health research should motivate a wider reevaluation of the evidence in relation to the theoretical frameworks.

### Alternate interpretations for empirical phenomena

Finding lifespan control in a robust theoretical model opens the door to interpreting a wide range of observations of aging-related phenomena as reflecting such an evolutionary response to natural selection. Such a factor does not preclude classic mechanisms such as mutation accumulation and antagonistic pleiotropy, but rather augments them, providing additional explanations that may contribute to observed phenomena. While necessarily speculative, here we suggest examples of cases where future study may find that selection for direct lifespan control plays a significant role. Since the model conditions essential to the qualitative result of self-limited lifespan—limited dispersal and limited resources—are typical features of natural systems, we would expect lifespan control to be a widely present phenomenon.

(1) The great range of natural lifespans observed among organisms, both dissimilar ones and those otherwise similar [6, 8], is traditionally explained as selection for longer life being insufficiently strong in the cases of shorter-lived organisms to extend their lifespans to match those of longer-lived ones. Instead or additionally, shorter lifespans could be actively selected for in some organisms, according to ecological conditions.

(2) Large single-gene or few-gene effects on lifespan [10] were initially surprising from the viewpoint of traditional theories [26]. Some have been interpreted as examples of antagonistic pleiotropy [22], where increased lifespan carries a fitness cost in early life [13]. A more direct interpretation is that they could represent effective means of control to regulate the tuning of lifespan for different circumstances.

(3) The evolutionary loss of mouthpart function in many adult insects, such as mayflies, is often the primary factor limiting lifespan [6]. The traditional explanation for this loss of function is evolutionary irrelevance after reproduction; instead, it could have come about through selection as a means of actively limiting lifespan. Similar mechanisms may be present in other animals: for instance, elephants go through six sets of molars in their lifetime, and starvation after the final set wears out may be the leading cause of death for older elephants [6].

(4) The model predicts that reproduction rate rises with increasing extrinsic mortality rate (Fig 4A). This is consistent with a field study of an island population of opossums, free from predators, which were observed to live longer and reproduce more slowly than mainland individuals [112]. These trends have been interpreted as evidence of increased senescence in the presence of increased predation [109, 112]. However, an alternate interpretation is that the shorter observed *natural* lifespan on the mainland is a direct result of the high predation (*intrinsic* lifespan is rarely observed there, as most opossums are killed by predators, with barely a quarter of females surviving to produce a second litter [112]), and the reproduction rate is higher to compensate.

(5) The model predicts the evolution of long lifespans for low consumption *v* relative to resource availability (Fig 2C, [77]). This is consistent with observations of animals such as marine fishes, which have longer lifespans and slower metabolisms at greater depths [113]; crocodilians, which go from weeks to years between meals [114] and are long-lived species characterized as exhibiting negligible senescence [115]; and cave animals, which, compared to surface dwellers, appear to have adapted to conditions of food scarcity by evolving significantly extended lifespan [116]. Similarly, populations limited by factors other than resource availability are predicted to evolve longer lifespans (e.g., as with the guppies subject to higher predation and having longer intrinsic lifespans [48]).

(6) The model demonstrates that in many circumstances, a strain exhibiting increased reproduction along with increased death rate has an advantage over slower-reproducing, longer-lived individuals. This is consistent with studies of cancer where rapidly replicating cells also have increased rates of apoptosis [117], suggesting an advantage over other possible cancerous types as well as normal cell populations.

(7) The model demonstrates an evolutionary advantage associated with reducing resource use through shortening lifespans. Other mechanisms for reducing resource use (particularly age-dependent) may have similar effects. For instance, metabolism typically declines with age [118], which may allow greater longevity while leaving more resources for descendants. A related issue is that of post-reproductive care of indirect offspring in humans (the “grandmother hypothesis”). A strong relationship exists between post-reproductive lifespan in women and their number of grandchildren [119]. (Note that a form of time-dependent fitness, measuring reproductive output at later generations, is necessary to describe this effect: there is no relationship between a woman’s lifespan and her number of direct offspring [119].) The benefits associated with grandmother care may compete against the benefits of increased resource availability through early death that we have focused on. In organisms where such care is present, its effects oppose selection for shorter lifespan, with potential relevance to the notably long lifespans of humans and perhaps some other primates [120–122].

### Time-dependent fitness

A useful characterization of the long-term behavior of evolutionary models is the time-dependent fitness [62, 63]: the average number of descendants of a given type at a given point in the future, not restricted to the next generation. This measure quantifies the long-term survival of the type including effects of changes caused by the type to its environment.

Looking at number of offspring as a function of generation number rather than time (Fig 6, right-hand plots) provides a connection to population growth rate in traditional theoretical treatments. If *x* is the generation number and *y*(*x*) is the number of invaders of that generation (y-value in the plot), then *y*(1) is the average number of direct offspring of an initial invader; similarly, *y*(*x* + 1)/*y*(*x*) gives the average lifetime reproduction per consumer in later generations. Dividing this quantity by the consumer lifetime, 1/(*v* + *rc* + *q*) (taking all causes of mortality into account), gives *r*, the average number of offspring per consumer per time step: $r=\frac{y(x+1)\cdot (v+q)}{y(x)-cy(x+1)}$. The population growth rate is this birth rate minus the death rate, *r* − (*v* + *rc* + *q*). However, as those plots show, an initial advantage in population growth rate does not necessarily characterize long-term reproductive success. The traditional single-generation measures of fitness and population growth rate are not sufficient descriptors of long-term success in spatial systems.

Note that this issue is distinct from a different way that temporal heterogeneity is more typically considered in evolutionary processes, which is in terms of a changing environment, such that a phenotype with a given fitness at one time (and hence in one environment) may confer a different fitness at another time (in another environment). The latter is distinct from the sense of time-dependent fitness (following previous work using this terminology [62]) we discuss here, in which a single organism’s fitness (considered in the classic sense of its number of offspring) has different values depending on the time horizon in the future at which the number of descendants is measured.

### Classic perspectives on lifespan control

Mainstream evolutionary theory considers it well-established that selection does not and cannot act in favor of decreased lifespan (unless more than compensated by a concomitant increase in early-life fitness, in the antagonistic pleiotropy framework [22]). A few characteristic quotations illustrate this point:

“The way evolution works makes it impossible for us to possess genes that are specifically designed to cause physiological decline with age or to control how long we live. Just as an automobile does not have a built-in plan for decline written in its blueprints, we do not possess genetic instructions that tell our bodies how to age or when to die.” [2]

“Evolutionary theory correctly asserts that aging is not an adaptive trait.” [25]

“There is a widespread but erroneous tendency to regard aging as programmed…. There are powerful arguments why [such a program] should not exist…. The suggested benefits from aging are ones that serve the interests of the species or group. Whenever a benefit at the *group* level is assumed to supersede the contrary interests of the *individual*, any evolutionary hypothesis must confront the problem of ‘cheating.’ Individuals in whom the aging program was inactivated by mutation would benefit from the sacrifice of others, while enjoying any fitness advantage that might accrue from immortality. Such mutations would therefore be expected to spread.” [26]

“Any hypothetical ‘accelerated ageing gene’ would be disadvantageous to the individual. It is therefore difficult to see how genes for accelerated ageing could be maintained in stable equilibrium, as individuals in whom the genes were inactivated by mutation would enjoy a selection advantage.” [24]

“Aging, a decline in condition with increasing age apparent as a reduction in survival and reproductive output, is apparently disadvantageous for the individual. Why then, does it exist at all? Is there some hidden advantage to aging? Evolutionary biologists tackled these questions early on and concluded that aging does not have a function and exists only because natural selection is less powerful late in life…. These two basic tenets, that aging is due to a declining force of natural selection and is not adaptive, still form the conceptual foundation of the biology of aging.” [123]

“There is a striking discrepancy between the diversity of theory on the evolution of senescence and its treatment in the literature. Empirical evaluations of the evolution of senescence focus almost exclusively on the classical theory, as do recent reviews.” [48]

These views can be traced back to Williams, who, in the article in which he proposed antagonistic pleiotropy, wrote: “Natural selection should ordinarily proceed towards lengthening life, not shortening it. Such selection, at the individual level, could conceivably be countered by selection at the population level, if senescence somehow favored group-survival…. The efficacy of such selection depends upon a rather complicated series of assumptions …A theory based on the simpler and more widely applicable principle of selection within a group would be preferable, unless the assumption of effective between-group selection proves to be necessary.” [22]

In this way Williams argued that selection for shorter lifespan should not be considered if any alternative explanation exists. Such a view risks blinding evolutionary biology to an important explanatory process, particularly with increasing evidence that selection above the individual level is an important force in evolution [62–64, 100–103, 124–128].

## Supporting information

S1 Video A successful invasion of consumers without intrinsic mortality by those with the capacity for programmed death. This video shows the full invasion of Fig 3, and illustrates characteristic spatiotemporal patterns for both types of populations.
(MP4)

S1 Table Consumers with the capacity for intrinsic mortality dominate those without it in invasion studies. This set of tables shows probabilities of successful invasions, for different values of resource growth rate *g* and consumption rate *v*, and for each combination of invaders and invaded having or lacking the capacity for intrinsic mortality.
(PDF)
