# Supplementary material for: Theory and associated phenomenology for intrinsic mortality arising from natural selection
Source: PLoS One. 2017 Mar 29;12(3):e0173677. doi: 10.1371/journal.pone.0173677 (PMC5371302; doi:10.1371/journal.pone.0173677)
Supplement: S1 Table — This set of tables shows probabilities of successful invasions, for different values of resource growth rate g and consumption rate v, and for each combination of invaders and invaded having or lacking the capacity for intrinsic mortality. (PDF) [file pone.0173677.s002.pdf]

Table S1. Consumers with the capacity for intrinsic mortality dominate those without it in invasion experiments.

$g = 0.05, v = 0.05$		Invaded	
		Immortal	Mortal
Invader	Mortal	$(2.51 \pm 0.05) \times 10^{-2}$	$(1.0 \pm 0.7) \times 10^{-5}$
	Immortal	$(2 \pm 1) \times 10^{-5}$	0

$g = 0.05, v = 0.1$		Invaded	
		Immortal	Mortal
Invader	Mortal	$(1.91 \pm 0.04) \times 10^{-2}$	$(1.1 \pm 0.3) \times 10^{-4}$
	Immortal	$(1.2 \pm 0.3) \times 10^{-4}$	0

$g = 0.05, v = 0.2$		Invaded	
		Immortal	Mortal
Invader	Mortal	$(1.23 \pm 0.04) \times 10^{-2}$	$(1.6 \pm 0.4) \times 10^{-4}$
	Immortal	$(2.8 \pm 0.5) \times 10^{-4}$	0

$g = 0.1, v = 0.05$		Invaded	
		Immortal	Mortal
Invader	Mortal	$(2.51 \pm 0.05) \times 10^{-2}$	$(2 \pm 1) \times 10^{-5}$
	Immortal	$(2 \pm 1) \times 10^{-5}$	0

$g = 0.1, v = 0.1$		Invaded	
		Immortal	Mortal
Invader	Mortal	$(2.38 \pm 0.05) \times 10^{-2}$	$(5 \pm 2) \times 10^{-5}$
	Immortal	$(1.4 \pm 0.4) \times 10^{-4}$	0

$g = 0.1, v = 0.2$		Invaded	
		Immortal	Mortal
Invader	Mortal	$(1.80 \pm 0.04) \times 10^{-2}$	$(1.1 \pm 0.3) \times 10^{-4}$
	Immortal	$(1.1 \pm 0.3) \times 10^{-4}$	0

$g = 0.2, v = 0.05$		Invaded	
		Immortal	Mortal
Invader	Mortal	$(2.24 \pm 0.05) \times 10^{-2}$	$(2 \pm 1) \times 10^{-5}$
	Immortal	$(3 \pm 2) \times 10^{-5}$	0

$g = 0.2, v = 0.1$		Invaded	
		Immortal	Mortal
Invader	Mortal	$(2.44 \pm 0.05) \times 10^{-2}$	$(5 \pm 2) \times 10^{-5}$
	Immortal	$(1.1 \pm 0.3) \times 10^{-4}$	0

$g = 0.2, v = 0.2$		Invaded	
		Immortal	Mortal
Invader	Mortal	$(2.12 \pm 0.5) \times 10^{-2}$	$(1 \pm 1) \times 10^{-5}$
	Immortal	$(7 \pm 3) \times 10^{-5}$	0

Probabilities of successful invasions for different values of resource growth rate g and consumption rate v (all with reproduction cost c=0), and for each combination of invaders and invaded having or lacking the capacity for intrinsic mortality ("mortal" and "immortal", respectively).
